# Primary Extra-Pleura Leiomyoma: A Case Report and Literature Review

**DOI:** 10.3390/curroncol29050240

**Published:** 2022-04-20

**Authors:** Asad Ullah, G. Taylor Patterson, Intisar Ghleilib, Islam A. Elhelf, Nikhil G. Patel, Nagla A. Karim

**Affiliations:** 1 Department of Pathology, Augusta University, Augusta, GA 30912, USA; aullah@augusta.edu (A.U.); ighleilib@augusta.edu (I.G.); npatel4@augusta.edu (N.G.P.); 2 Medical College of Georgia, Augusta, GA 30912, USA; gpatterson1@augusta.edu; 3 Department of Radiology, Augusta University, Augusta, GA 30912, USA; ielhelf@augusta.edu; 4 Department of Medicine, Hematology and Oncology, Augusta University, Augusta, GA 30912, USA

**Keywords:** pleural tumor, leiomyoma, computed tomography

## Abstract

Leiomyomas are a common type of benign soft tissue tumor arising from smooth muscle, most often occurring within females’ genitourinary and gastrointestinal tract. However, primary leiomyomas of the chest wall residing in the extra-pleural space are an extremely rare subset of leiomyomatous lesion presentation. We present a case of a fifty-two-year-old male who initially presented complaining of dyspnea worsening with exertion. Computed tomography imaging was performed showing an extra-pleural mass residing under the left sixth rib. Subsequent core needle biopsy and immunohistochemical staining were performed, and the definitive diagnosis of primary leiomyoma of the posterior mediastinal chest wall. Although extremely rare, this neoplastic condition should be included in your differential diagnosis when diagnostic imaging reveals a benign mass residing in the extra-pleural space, and subsequent biopsy specimens consist of smooth muscle fibers.

## 1. Background

Primary neoplasms arising in the pleural and extra-pleural space are an abnormal finding since 75% of tumors found in this area can be attributed to metastases from around the body, with the remaining 25% being attributed to malignant pleural mesothelioma and solitary fibrous tumors [1,2]. Therefore, extra-pleural leiomyoma are seldom reported in medical literature. The condition is characterized by the growth of neoplastic smooth muscle tissue within the extra-pleural space [1,3]. Here, we present a case of primary leiomyoma of the chest wall arising in a 52-year-old male to expand the medical literature about this rare condition. In addition, a literature review of previously published cases about this disease state is performed.

To our knowledge, this is the most comprehensive and up-to-date review of primary pleural leiomyoma cases.

## 2. Case Report

**Figure 1:**
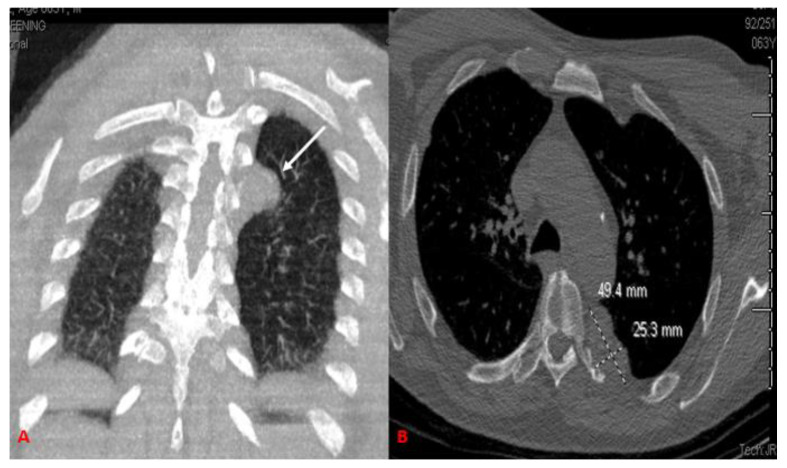
Non-contrast CT scan of the chest. Coronal (**A**) images show a well-circumscribed lobulated, homogeneous extrapleural mass (arrow) adjacent to the left 6th rib posteriorly. Bone window (**B**) shows subtle erosive changes at the left costovertebral junction adjacent to the mass. The homogeneous mass completely surround costovertebral junction.

**Figure 2:**
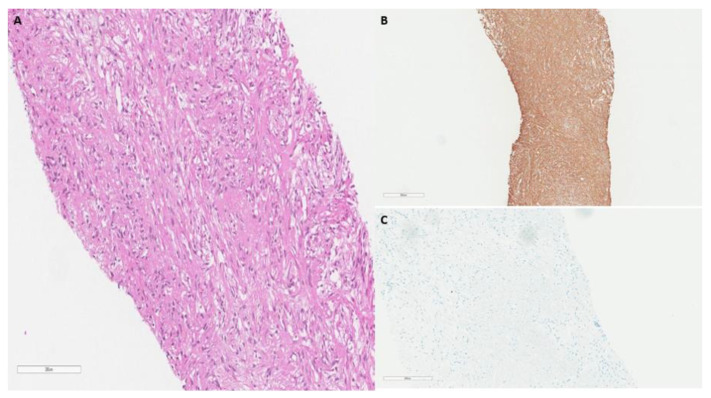
(**A**) H&E Pathological findings from CT guided needle biopsy show fascicular smooth muscle bundles separated by vascularized connective tissue, small round nuclei with abundant eosinophilic cytoplasm (20×). (**B**) SMA stain: strong cytoplasmic staining. (**C**) Keratin stain: negative keratin staining.

In June 2015, a fifty-two-year-old male presented to the clinic with a chief complaint of several episodes of dyspnea on exertion. His past medical history was notable for mental retardation, schizophrenia, hepatitis C and chronic obstructive pulmonary disease (COPD). He also has a history of tobacco abuse and currently resides in a nursing home facility. A chest radiograph was performed showing scattered granulomas with kyphosis and compression deformities of the thoracic spine. A year later, and with no resolution of symptoms, a non-contrast CT scan of the chest was scheduled. The patient exhibited a unilateral extra-pleural mass residing over the posterior portion of the left 6th rib, measuring 4.9 × 3.9 × 2.5 cm along its maximum anteroposterior, transverse, and craniocaudal dimensions, respectively. In addition, subtle erosive changes were noted in the adjacent costovertebral junction (Figure 1). A CT-guided needle core biopsy of the left, extra-pleural mass was obtained. Histological analysis showed intersecting fascicles of bland spindle cells with abundant eosinophilic cytoplasm. No high-grade morphologic features such as brisk mitosis, overt atypia, or necrosis were noted. Immunohistochemical staining showed the lesion was strongly positive for smooth muscle markers (SMA) and desmin and was negative for keratin, CD34, and human melanoma black 45 (HMB45) (Figure 2). The final diagnosis of primary leiomyoma of the lung was made. The mass was removed by a minimally-invasive procedure. After the removal of the mass, the patient’s symptoms improved clinically. The patient’s case was discussed at the multidisciplinary thoracic tumor board. Close clinical and radiologic follow-up was recommended. Upon returning for a 6-month follow-up, his symptoms had resolved completely, and he has continued to remain in good health.

## 3. Literature Analysis

Primary extra-pleural leiomyoma of the lung is an extraordinarily rare event, with only 14 cases being reported in medical literature to date. Therefore, a comprehensive literature analysis was conducted on PubMed with the keywords “pleural primary leiomyoma” as search criteria. Twelve manuscripts, reporting on fourteen patients, were discovered and determined eligible for analysis. The patient reported on in this manuscript was included as well (Table 1).

## 4. Discussion

Leiomyomas are benign smooth muscle tumors that were first discovered and characterized in 1854 [3]. Most cases occur in women of childbearing age, with leiomyomas representing the most common neoplasms of the uterus, affecting 20–30% of women between the ages of 30 and 50 years [14,15,16]. However, although uncommon, these smooth muscle neoplasms have been found to occur in other organs such as the small intestines, esophagus, and lung parenchyma [3,4]. Moreover, leiomyomas arising within the pleura and extra-pleural space of the lungs are an extremely rare event, with only 14 reported cases noted in medical literature to date (Table 1). Our literature analysis found the average age of diagnosis was around 41 years old, which is fairly similar to the mean age of 35 for diagnosis of uterine leiomyoma. Of the 15 reported cases, 33% were reported to occur in males while the remaining 66% were reported in female patients.

The majority of individuals with leiomyoma are asymptomatic for many years and only diagnosed incidentally upon physical exam or during imaging for other non-related problems [17,18]. Of the 15 cases used in our review, data about the initial presenting symptoms were found in 14 patients. Of those, 7 of 14 were found to be asymptomatic at diagnosis. The remaining 7 cases all had respiratory complaints, with chest pain and cough being the most common.

Pleural leiomyoma is a benign smooth muscle tumor with low malignant potential. It has the potential for local invasion into surrounding structures. The increased size of the tumor with the involvement of mediastinum makes it difficult to complete resection. It has been reported that pleural leiomyoma can disseminate through the needle tract of FNA biopsy. Thus, an FNA biopsy should be avoided if the tumor can be completely resected [5].

The diagnosis is mainly based on diagnostic imagining and histologic analysis. Typical radiographic findings on CT scans include a well-encapsulated, solitary lesion ranging from a few millimeters to several centimeters, with minimal invasion of adjacent structures [5]. Of the patients included in the review, 12 presented with a solitary lesion diagnosed via CT, while 2 cases had multiple masses at initial diagnosis. However, a definitive diagnosis of primary extra-pleural leiomyoma is difficult and can only be confirmed in conjugation with histological and immunohistochemical analysis [19]. Typical histologic features show negative signs of malignancy including, the absence of elevated mitotic count, low cellularity, lack of cytologic atypia with pleomorphism, and prominent fibrosis, with these features being confirmed through the use of H&E staining [5]. In addition, the neoplastic cells must be of smooth muscle origin and thus should stain positive for smooth muscle actin and desmin [4,5]. The absence of malignant histologic features noted upon H&E staining, combined with positive staining for SMA and desmin provides unambiguous evidence for the diagnosis of leiomyoma.

The role of PET/CT in precision medicine is an essential part of determining the staging of disease, recurrence, residual disease, and evaluating the response of patients to therapy. Combining PET with CT provides high sensitivity with PET camera for tracer distribution while CT provides anatomic localization with precision, thus making PET/CT a sensitive and a more accurate modality for evaluating malignancies [20].

No definitive treatment guidelines have been established for this condition; however, the general practice of complete surgical resection is preferred due to the well-defined and minimally invasive nature of the tumor. Thirteen of the fifteen patients elected this route of treatment (Table 1). Although in general, leiomyomas have low malignant potential, surgery is justified in these specific cases due to the chance of future tumor growth, leading to compression and invasion to adjacent structures and ultimately to serious symptoms. Overall, the prognosis for extra-pleural leiomyoma is excellent as evidenced by the 0% recurrence rate seen in Table 1 with as much as 5 years post-surgical intervention.

## 5. Conclusions

Primary extra-pleural leiomyoma is an extremely rare neoplastic proliferation of smooth muscle origin arising in the extrapleural cavity of the lungs. Diagnostic imaging and pathological analysis are essential for distinguishing from other pleural cancers such as mesothelioma and solitary fibrous tumors, as well as other leiomyomatous lesions of the lung. Overall, the condition has an excellent prognosis with the vast majority of patients electing for complete surgical resection with little evidence to suggest future recurrence. In conclusion, primary extra-pleural leiomyoma should be included in your differential diagnosis when an extra-pleural mass is found on diagnostic imaging, and subsequent histological analysis reveals a specimen absent of malignant features and exhibiting smooth muscle fibers.

## Figures and Tables

**Table 1:** Clinical features of reported extra-pleural leiomyoma cases.

Author	Age/Sex	Chief Complaint	Size (cm)	Treatment	Outcome
Nakada et al. [4] (2013)	28/F	Chest pain	4.2 × 3.3 × 3.2	Complete surgical resection	2-month follow-up, no recurrence
Qiu et al. [5] (2011)	45/M	Chest pain	5.9 × 8.0 × 6.2	Complete surgical resection	15-month follow-up, no recurrence
Rodríguez et al. [1] (2010)	48/F	Pleuritic chest pain	18 × 14 × 11	Complete surgical resection	18-month follow-up, no recurrence
Arikura et al. [6] (2011)	52/F	N/A	6.5 × 5.5	Complete surgical resection	N/A
Tanaka et al. [7] (2000)	40/F	Asymptomatic	3.5 × 3.0	Complete surgical resection	No recurrence noted
Moran et al. [8] (1995)	21/F	Asymptomatic	Fragments of varying size	Observation	4-month follow-up, alive
Moran et al. [8] (1995)	23/F	Asymptomatic	10 × 9 × 5.5	Observation	6-month follow up, alive
Proca et al. [9] (1995)	32/M	Asymptomatic	4.3 × 7.0	Complete surgical resection	12-month follow up, alive with no recurrence
Turhan et al. [10] (2008)	50/F	Chest pain	4.0 × 4.0	Complete surgical resection	53-month follow up, alive with no recurrence
Ziyade et al. [11] (2011)	33/F	Chest pain and heartburn	5.3 × 4.0 × 3.4	Complete surgical resection	14-month follow up, symptom-free with no recurrence
Nose et al. [12] (2006)	55/F	Asymptomatic	1.5 × 1.5	Complete surgical resection	26-month follow up, symptom-free with no recurrence
Haratake et al. [3] (2016)	42/F	N/A	15.0 × 12.0	Complete surgical resection	60-month follow up, symptom-free with no recurrences
Kuman et al. [13] (2014)	32/F	Chest pain	N/A	Complete surgical resection	45 month follow up, symptom-free with no recurrences
Kuman et al. [13] (2014)	43/M	Chest pain	2.0	Complete surgical resection	40-month follow up, symptom-free with no recurrences
Present case (2022)	52/M	Dyspnea on exertion	4.9 × 3.9 × 2.5	Complete surgical resection	6-month follow up, symptom-free with no recurrences

## Data Availability

No new data were created or analyzed in this study. Data sharing does not apply to this article.
